# Supplementary material for: GPR161–GLI3 repressor signaling at cilia directs apical constriction and cell fate during cranial neural tube closure
Source: Development. 2025 Dec 19;152(24):dev205171. doi: 10.1242/dev.205171 (PMC12752497; doi:10.1242/dev.205171)
Supplement: Supplementary information [file develop-152-205171-s1.pdf]

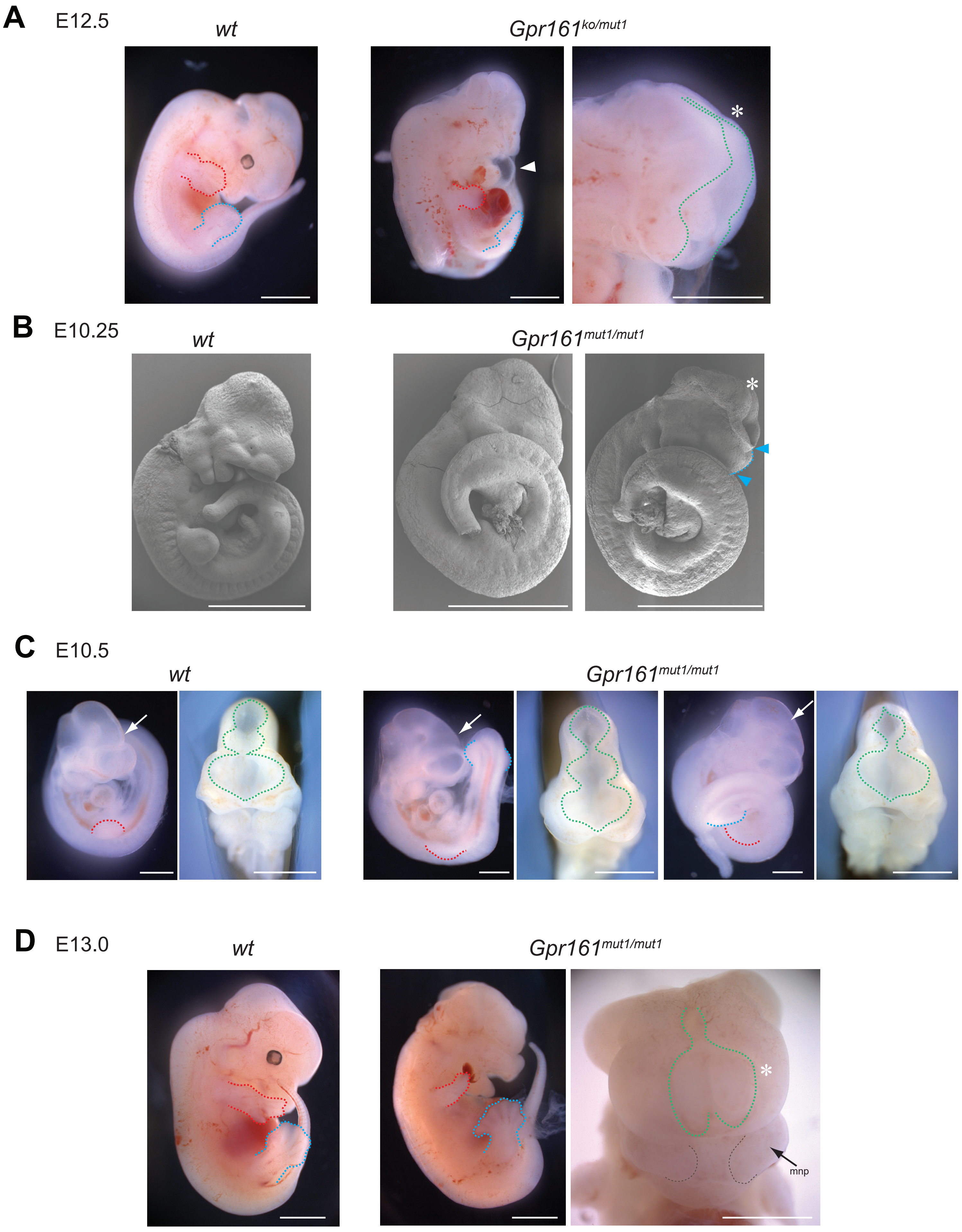

Development • Supplementary information

Fig. S1. Exencephaly in *Gpr161* mutant mice.

(**A**) Whole mount images of wild-type (*wt*) and *Gpr161 ko/mut1* littermate embryos at E12.5. showing that the latter do not have optic cups and exhibit fully open cranial neural tube. Asterisk, exencephaly. Anterior neuropore closure defect is marked by green dotted lines. Also note pericardial effusion in *Gpr161 ko/mut1* (arrowhead).

(B) SEM images of representative control, *Gpr161 mut1/mut1* littermate embryos at E10.25. One *Gpr161 mut1/mut1* embryo showed exencephaly (asterisk) with anterior neuropore closed (arrowheads).

(C) Whole mount images of wild-type and *Gpr161 mut1/mut1* littermate embryos at E10.5 shows widened anterior neural tube in *Gpr161 mut1/mut1* despite cranial closure. Lateral views and zoomed *en face* images are shown. Arrows show apposed cranial neural folds. Structurally widened anterior neural tube is shown in *Gpr161 mut1/mut1* compared to wild-type by green dotted lines.

(**D**) Whole mount images of control and *Gpr161 mut1/mut1* littermate embryos at E13, showing that the latter exhibit exencephaly and widely spaced medial nasal processes (mnp). Asterisk, exencephaly. Anterior neuropore closure defect is marked by green dotted lines. Medial nasal processes are marked by grey dotted lines.

Scale: (A) 2 mm; (B) 1 mm; (C) 2 mm.

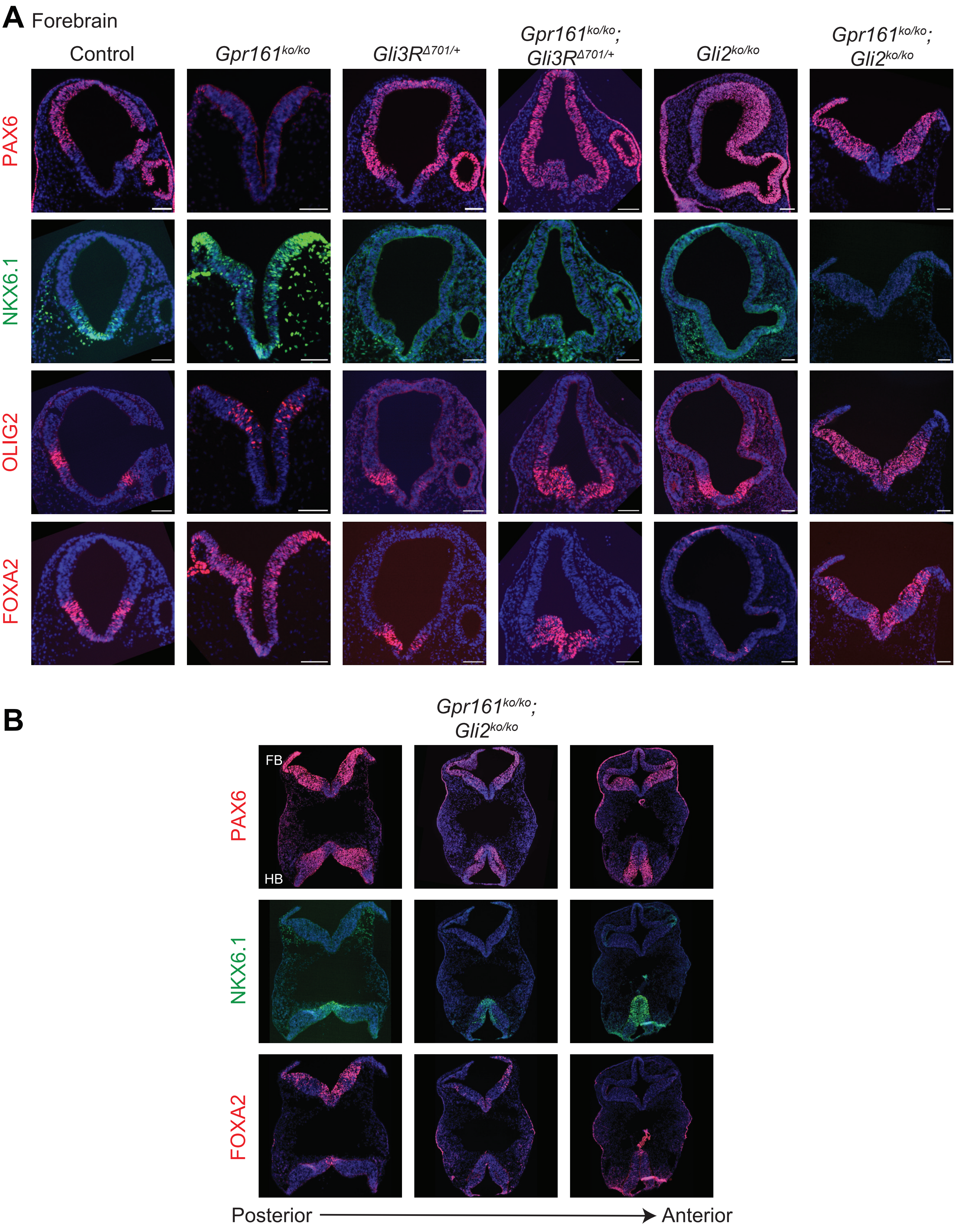

Fig. S2. Floor plate marker ventralization in E9.25 *Gpr161* ko forebrain is selectively rescued by *Gli3R* expression but not from *Gli2* deletion.
(**A**) Panels showing forebrain cranial horizontal sections immunostained using designated markers for embryos as in Figure 5 dissected at E9.25. All images are counterstained with DAPI.**(B)** Rostro caudal serial cranial sections of *Gpr161* ko/ko; *Gli2* ko/ko embryo in Fig. 5 is shown. Note posteriorly open and anteriorly closed forebrain regions. Note ventralized FOXA2 in both open and closed forebrain regions. Abbreviations: FB, forebrain; HB, hindbrain

Development • Supplementary information

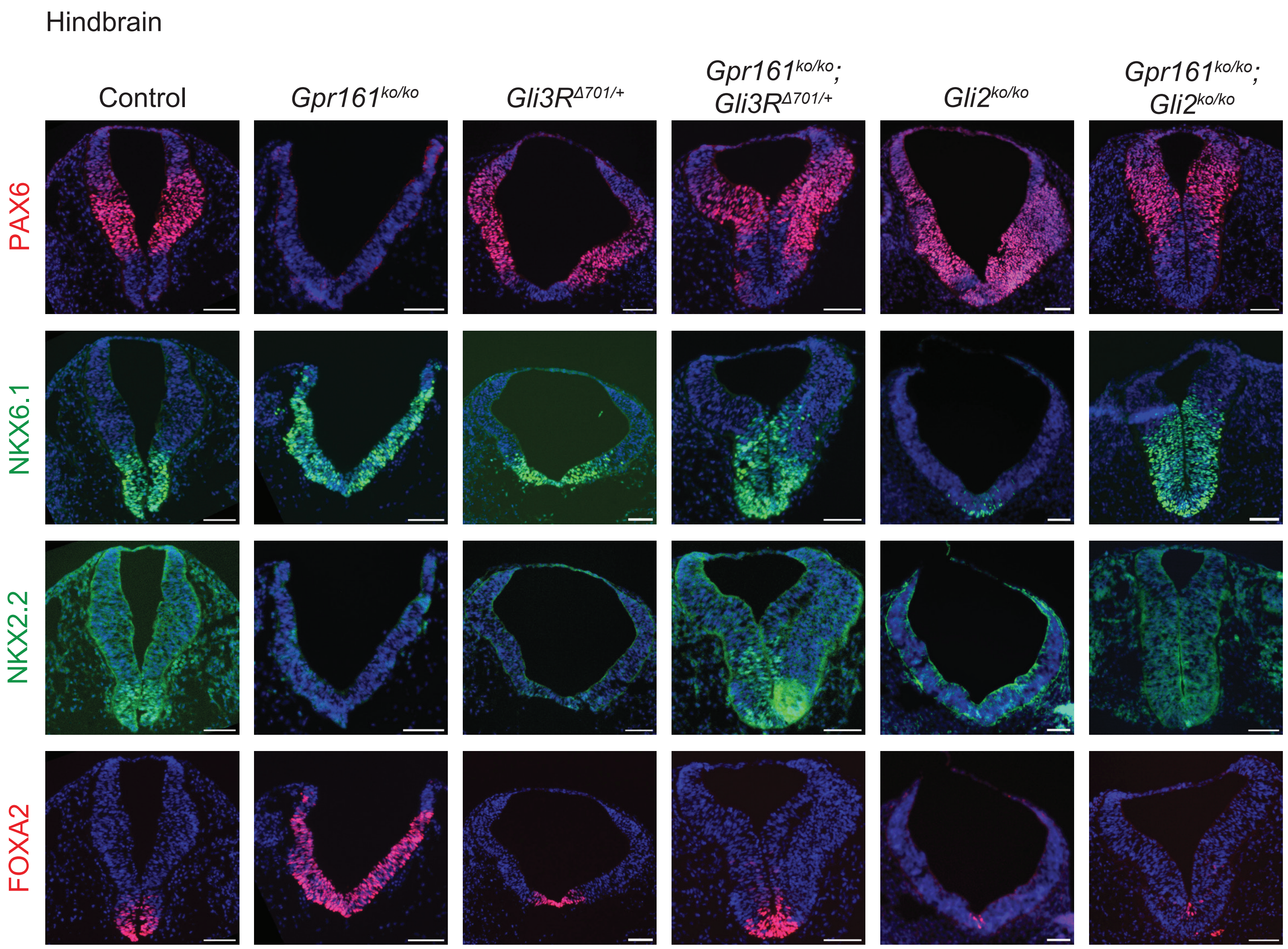

Fig. S3 Floor plate marker ventralization in E9.25 *Gpr161* ko hindbrain is rescued by *Gli3R* expression or *Gli2* deletion.

Panels showing hindbrain cranial horizontal sections immunostained using designated markers for embryos as in Fig. 5 dissected at E9.25. All images are counterstained with DAPI.

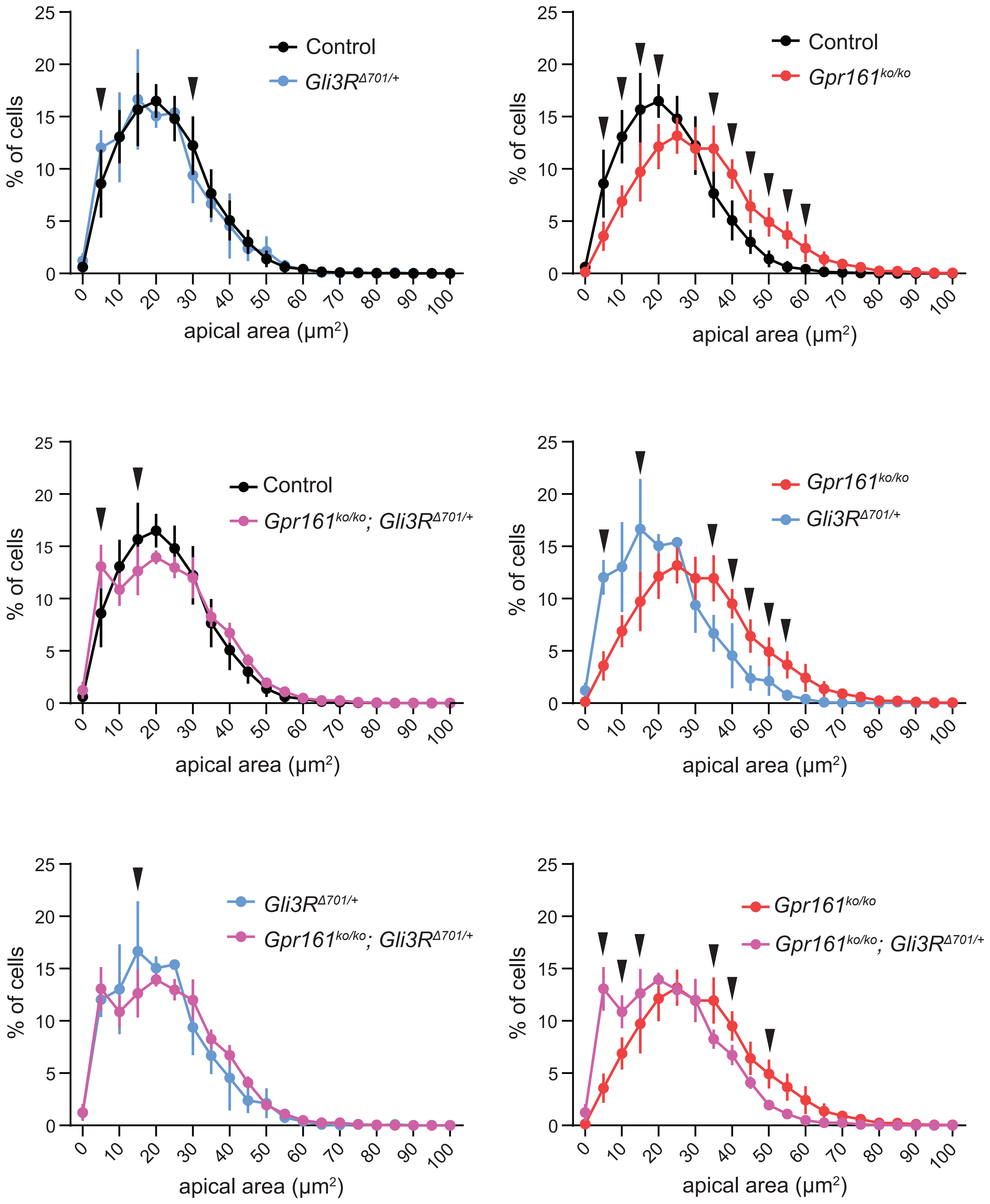

Development • Supplementary information

Fig. S4. *Gli3R* expression rescues midbrain apical constriction defects in *Gpr161* mutant embryos.

The full set of pairwise comparisons of apical area distributions from Fig. 8C are shown. All data shown as mean ± s.d. Arrowheads indicate area bins that differ significantly ($p < 0.05$) between the two distributions according to a two-way ANOVA with Sidak's correction for multiple comparisons. Note that there are two or fewer bins that differ between control and *Gli3R*$^{\Delta 701/+}$, control and *Gpr161 ko/ko Gli3R*$^{\Delta 701/+}$, and *Gli3R*$^{\Delta 701/+}$ and *Gpr161 ko/ko Gli3R*$^{\Delta 701/+}$ indicating good agreement between these distributions. Conversely comparison of *Gpr161 ko/ko* alone with any of the other distributions results in a minimum of six bins showing significant differences, with *Gpr161 ko/ko* distribution having a uniformly rightward shift toward larger cell areas compared to all other conditions.

Development • Supplementary information
